# Core executive functions are associated with success in young elite soccer players

**DOI:** 10.1371/journal.pone.0170845

**Published:** 2017-02-08

**Authors:** Torbjörn Vestberg, Gustaf Reinebo, Liselotte Maurex, Martin Ingvar, Predrag Petrovic

**Affiliations:** Department of Clinical Neuroscience, Karolinska Institutet, Stockholm, Sweden; Universita degli Studi di Verona, ITALY

## Abstract

Physical capacity and coordination cannot alone predict success in team sports such as soccer. Instead, more focus has been directed towards the importance of cognitive abilities, and it has been suggested that executive functions (EF) are fundamentally important for success in soccer. However, executive functions are going through a steep development from adolescence to adulthood. Moreover, more complex EF involving manipulation of information (higher level EF) develop later than simple executive functions such as those linked to simple working memory capacity (Core EF). The link between EF and success in young soccer players is therefore not obvious. In the present study we investigated whether EF are associated with success in soccer in young elite soccer players. We performed tests measuring core EF (a demanding working memory task involving a variable n-back task; dWM) and higher level EF (Design Fluency test; DF). Color-Word Interference Test and Trail Making Test were performed on an exploratory level as they contain a linguistic element. The lower level EF test (dWM) was taken from CogStateSport computerized concussion testing and the higher level EF test (DF) was from Delis-Kaplan Executive Function System test battery (D-KEFS). In a group of young elite soccer players (n = 30; aged 12–19 years) we show that they perform better than the norm in both the dWM (+0.49 SD) and DF (+0.86 SD). Moreover, we could show that both dWM and DF correlate with the number of goals the players perform during the season. The effect was more prominent for dWM (*r* = 0.437) than for DF (*r* = 0.349), but strongest for a combined measurement (*r* = 0.550). The effect was still present when we controlled for intelligence, length and age in a partial correlation analysis. Thus, our study suggests that both core and higher level EF may predict success in soccer also in young players.

## Introduction

A skilled and successful soccer player needs to process a large amount of information in a short time under mental pressure. Many decisions must be made fast and quickly and be reevaluated depending on the demand on the pitch. The necessary behavior includes a creative decision-making in which both accuracy and speed are at top level. Such behavior helps the soccer player to “read the game” and make successful a priori expectations [1]. These cognitive abilities are called game intelligence in soccer [1]. In psychology similar cognitive abilities are called executive functions [2–4]. Executive functions (EF) is used as a term to describe cognitive processes that regulate thought and action, especially in non-routine situations [5]. Examples of such processes as defined in cognitive psychological terms are problem solving, planning, sequencing, selective and sustained attention, inhibition, utilization of feedback, multi-tasking, cognitive flexibility and ability to deal with novelty [6, 7].

Previously, research has focused on sport specific perceptual abilities that are important for successful sport behavior and that have been able to distinguish between elite and novice [8–10]. While perceptual abilities often include some core EF the definition is wider and includes several non-EF aspects of perceptual capacity. EF may pinpoint specific underlying cognitive process even on a brain function level [11, 12]. Moreover, sport tests of perceptual functions have often been designed for a specific sport or study [8–10]. In contrast, EF may more easily be compared over sports and with the general population since they are standardized. This makes it possible to draw general conclusion from the tests, i.e. if some cognitive abilities are more important for specific sports or positions in a team.

In our previous study [13], we showed that senior elite and semi-elite soccer players had significantly better measures of different EF in comparison with the standardized norm group for both men and women. Moreover, the elite players outperformed the semi-elite players in these tests, and EF capacity was shown to predict successful performance in terms of goals and assists the following two years. The main results have been confirmed in a study where adult elite players in soccer, ice hockey and rugby performed significantly better than amateur athletes and PhD-students in an EF demanding task [14]. The test that was used included scanning ability, attention, cognitive flexibility, multi-processing, working memory, and inhibition [15, 16]. A recently published study has observed similar result in elite ice hockey players using a similar set of tasks as in the present study [17].

A main question is whether similar results may be observed in children and adolescent, where EF have not fully developed. The general results above have been extended to children and adolescent players in two recent studies [18, 19] indicating better EF in elite vs. non-elite junior soccer players. However, it is not known whether EF in junior elite soccer players are above the mean for a normal population nor whether they actually predict successful behaviour—as we have previously shown for adults [13].

EF are dependent on different prefrontal structures [20]. Since the prefrontal lobes mature slowly and are not considered to be fully developed until mid-twenties [21, 22] EF also develop gradually during many years. Functions like attentional control, processing speed, cognitive flexibility, goal setting, response inhibition, and working memory mature through late childhood and into adolescence to be fully developed around 19 years of age [23–26]. Strategic planning and the organization of goal-directed behavior seem to reach a top capacity between the 20 and 29 years of age [27]. EF may be divided into *Core EF* (CEF) and *Higher level EF* (HEF) [28, 29]. Working memory, cognitive flexibility and inhibitory control can be defined as CEF [29]. EF tasks involving reasoning, problem solving, and planning can be defined as HEF [29]. The distinction between CEF and HEF is building on the demands of the task [28]. For example, holding information on-line in order to make a simple decision is more related to CEF. Conversely, maintaining and manipulating information in order to strategically organize goal-oriented behavior is more related to HEF. The level of CEF develops to its full capacity earlier in the lifespan than HEF, mostly before early adolescence [28, 30, 31]. Considering such distinction in the development of different EF, HEF should play a minor role in childhood and in early adolescence for success in soccer compared to physical advantage (like length, strength and speed) and basic ball control skills—but be more important in adulthood. In contrast, CEF should have an impact for success also in earlier age.

A study of EF in young soccer players [18] included eighty-four elite and forty-two age-matched amateur soccer players aged 8 to 16 years and tested general executive functions including the Stop Signal task (motor inhibition), the Attention Network Test (alerting, orienting, and executive attention) and a visuo-spatial working memory task. This study showed that elite junior soccer players outperformed amateur junior players in suppressing ongoing motor responses (inhibition) and in the ability to attain and maintain an alert state. No group differences were found for orienting, executive attention and visuo-spatial working memory. Thus, while these results pointed out that there are differences in EF capacity between elite players and amateurs already in young ages, the study did not report whether the elite players were better than the population in general nor whether the difference also predicted a more successful behavior as a player.

Recently, another study investigated the relationship between general executive functions and performance level on eighty-eight elite and sub-elite youth soccer- players aged 13–17 years [19]. The study reproduced our previous findings [13] that elite soccer players were better in design fluency, an EF measure that includes speed, scanning ability, cognitive flexibility, inhibition, creativity and working memory. Apart from Design fluency also Stop-signal reaction task and Trail making test were shown to be better performed in young elite soccer players [19]. This study also focused on the difference between capacity for “lower-level” cognitive processes (including reaction time and visuo-perceptual abilities) and “higher-level” cognitive functions (including working memory, inhibitory control, cognitive flexibility, and “metacognition”)—mirroring the division between CEF and HEF to a certain degree. A drawback with the study was that it did not test whether the results were related to soccer playing behaviors. Thus, the study could not state whether EF capacity was predictive on success measures.

Although the studies presented above suggest that EF are important already before adulthood in soccer it is still not known how young elite soccer players score in EF as compared to the norm population, nor if the result predicts success in soccer—as has been suggested for senior soccer players [13]. We adapted a similar model from our previous study by comparing the general EF capacity of young players and success factors like goals and assists. However, as the complexity in the game on the field is not as advanced for children and adolescents as for senior players we suggested that the relations would be more related to goals and only to a lesser degree to assists. We hypothesized that the scoring goals relation is stronger for CEF than for HEF in young elite soccer players due to the fact that more advanced cognitive functions have developed less before adulthood.

## Methods

### Ethics statement

The study was approved by the local ethical committee (Regionala etikprövningsnämnden i Stockholm; Dnr: 2013/1976-31/3) and was performed in full compliance with the Declaration of Helsinki. All subjects were given verbal and written information on the study and gave their verbal and written informed consent to participate. Players below 16 years of age needed consent from a guardian while older players gave written consent themselves. Consent was also obtained from the club.

### Participants

Forty-nine top youth soccer players were recruited from an elite soccer academy in Sweden. The participants were boys born 1994 to 2001. Their teams were playing at the top level of their respective age group. Based on the academy’s statistics from the study period February 2012 to February 2014 thirty of these players scored at least one goal in a game. These players were defined as the test group for our analysis (n = 30; age range 12 to 19 years; mean age = 14.93 years)—See Figures A and B in S1 File for age distribution.

### Materials

In the present study we used tests from two test batteries for our main analysis: *CogStateSport computerized concussion testing* (CS; re-brand as Axon Sport) [32, 33] and *The Delis-Kaplan Executive Function System test battery* (*D-KEFS)* [34–36]. All test included in the present study have been standardized to a normal population of different age spans (See S1 File).

#### CogStateSports (CS)

**CS** is a non-verbal psychomotor test battery that measures attention, cognitive process speed, decision-making, speed and accuracy of short-term memory and encoding of working memory [32, 33]. The subjects are shown different play cards on a computer screen and have to react as fast and correct as possible in the various tests using different key responses. In the first test (“Processing speed”), measuring simple response time, the subject has to respond to any card that is displayed. In the second test (“Attention”), measuring simple attention, the subject has to respond whether the card is red or black. In a third test (originally denoted “Working memory”), measuring simple working memory, the subject has to decide if the previous card is the same as the card before (i.e. one back memory-test). In a fourth test (originally denoted “Learning” in CS) the subject has to respond if he has seen the displayed card any time earlier in the test sequences—a measure of more demanding working memory. In the present study we call this test **demanding Working Memory (dWM)**. We chose this test as the main test in our study to capture the player ability for CEF since no cognitive manipulation is needed in order to solve it. Moreover, the test is more demanding than the one back memory-test and will better mirror the cognitive challenge on the pitch where there is a constant flood of information and events from different time points must be processed.

#### D-KEFS

***D-KEFS*** is a test battery measuring different aspects of EF and the subtests used in this study was ***Design Fluency***, ***Colour-Word Interference Test*** and ***Trail Making Test***. It is routinely used in clinical assessments of patients and there are well-described norms for the general population [34, 37, 38].

**Design Fluency (DF)**, is a standardized test which measures on-line multi-processing such as creativity, response inhibition, and cognitive flexibility [35, 36] and thus simulates the executive chain of decision making in a similar way as in a real soccer situation. *DF* is also a non-verbal psychomotor test in which the participant uses a pen to combine dots in a square with four lines. In *Condition 1* the task is to find as many different combinations as possible of binding together filled (black) dots under time pressure (60 sec) and the participant is not allowed to use a solution twice. The participant needs to remember previous responses in an online working memory and update new rules accordingly (i.e. not repeat previous combinations). He or she must use inhibition skills in order not to repeat previous responses. The participant also needs to constantly use a scanning skill to find new solutions to fulfill the task. In *Condition 2* unfilled dots have been added to the square, and the task is to combine them with lines as in Condition 1. The filled dots are still present but the participant is not allowed to use them in the task. In *Condition 3* both filled and unfilled dots are present. The task is to connect lines as above but also to constantly switch between a filled and an unfilled dot. A combination score (*Total Correct Score*) of all three subtests of *DF* were used as previously [13] to capture both more “simple creativity” and “advanced creativity” with a higher demand on both inhibition and cognitive flexibility—mirroring the variability of problem solutions needed on the pitch. As this test consists of several levels of information manipulation it represents a form of HEF. However, at the same time it is not dependent on language skills.

As previously [13], we used additional EF tests including *Colour-word interference test* (CWI) as well as *Trail making test* (TMT) as exploratory tests. *CWI* is a *Stroop* test depending on verbal inhibition. In the present study we used the combination of the subtests *Condition 1* and *2* (tests that measures cognitive flexibility and to some extent control for reading ability) and *Condition 3*, (*CWI-3*) which includes a high degree of inhibition and represents the classical form of the Stroop task. From TMT we used the combination of subtest *Condition 2* and *3* (tests that combines scanning ability with short-term memory) and *Condition 4* (a test that combines scanning ability, cognitive flexibility, multi-processing and short-term memory). These tests measure general executive functions, including a language aspect, without the creativity or problem solving abilities aspects important in *DF*. Especially, the influence of reading capacity deems CWI and TMT as not perfect EF tests in a group of young individuals where the degree of reading knowledge may be highly variable. Therefore, they are not optimal in the present analysis but serve as a control to the main test [13].

#### Composite measurement

In order to get a more general EF-measurement consisting of both CEF and HEF components we also calculated a composite measurement by adding up the scale score transformed to z-score from *DF-Total Correct*, and d*WM* (denoted as *Composite measurement*).

#### IQ

Raven’s Standard Progressive Matrices [39] was used to approximate the average IQ of the test group (Table SPM 9, Smoothed Summary Norms for Children and Young People in the United States of America [39]). Raven’s Standard Progressive Matrices is a non-verbal intelligence test developed as a cognitive test for different cultural and socioeconomic groups that can be used world-wide [40, 41] in order to capture general intelligence or *g* [42].

### Procedure

The participating players were tested at a facility next to the academies training ground from June till October 2013. One experimental leader tested the players in a standardized process (See S1 File). Data concerning of how many goals and assists the players performed in average per game were delivered from the soccer academy’s statistic system. We followed the performance of players from February 2012 till February 2014.

### Statistical analysis

Data were analyzed using IBM SPSS Statistics 23.0.0. Kolmogorov-Smirnov test was used to test distributions for normality. One sample T-test was used to compare the cognitive test result of the group with the normal population.

The Pearson product-moment correlation coefficient was used to explore the relationship between the players cognitive test result and outcome of scored goals (and assists).

In the partial correlation tests, we assessed the relation between the result of the players’ cognitive tests and the number of goals (and assists) they made after controlling for length, year of birth and IQ.

Since the EF tests show a high degree of correlation between each other and we only used two tests for testing our main hypothesis we did not correct for number of tests performed. However, such a correction would not change the main results.

## Results

### Descriptive tests

#### Lower level of cognition

The soccer players performed 0.9 SD (normed mean: 100; SD = 10) above the normal population in the *Process speed* scaled scores (t(29) = 15.392, *p* = .000; two-tailed t-test) and 0.84 SD (normed mean: 100; SD = 10) above the normal population in the *Attention* scaled scores (t(29) = 13,461, *p* = .000; two-tailed t-test). However, there was no significant relationship between the player’s results on *Process Speed* and the number of made goals (*r* = .135; *p* = .230; one tailed correlational test) nor between the results on *Attention* and the number of made goals (*r* = .099; *p* = .295; one tailed correlational test).

#### IQ

The average IQ of the test group using Raven’s Standard Progressive Matrices was estimated to the 45.93. percentile (SD 24.6; Range 10–90). The groups result did not differ from the norm (t(29) = -0.407,; p > .05).

### Development of EF

#### DF

There was a significant correlation between the year of birth and the result on *DF-Total Correct* using raw score and number of correct figures (*r* = -.318; *p* = .043, one tailed correlational test).

#### dWM

There was no significant relationship between the player’s year of birth and the result on dWM using raw score of accuracy (*r* = -.174; *p* = .179; one tailed correlational test).

#### CWI

There was a significant relationship between the player’s year of birth and the result on CWI 1–2 using raw score and time in seconds (*r* = .312; *p* = .046, one tailed correlational test). There was also a significant relationship between the player’s year of birth and the result on CWI-3 using raw score and time in seconds (*r* = .361; *p* = .025, one tailed correlational test).

#### TMT

There was a significant relationship between the player’s year of birth and the result on TMT2-3 using raw score and time in seconds (*r* = .317; *p* = .044 one tailed correlational test). There was a non-significant tendency of a relationship between the player’s year of birth and the result on TMT-4 using raw score, time in seconds (*r* = .255; *p* = .087 one tailed correlational test).

### Hypothesis testing

Our specific hypothesis pertained to the results from the dWM and DF tests. The data gathered from these tests did not deviate from the normal distribution accordingly to Kolmogorov-Smirnov test.

#### Cross-sectional tests

***dWM*:** The soccer players performed 0.49 SD (normed mean: 100; SD = 10) above normal population in the *Learning* scaled scores (t(29) = 3.376, *p* = .002; two-tailed t-test).

***DF*:** The soccer players performed in average 0.86 SD *scores* (normed mean: 10; SD = 3) above normal population for their age using the *Total Correct* score (t(29) = 5.501, *p* = .000; two-tailed t-test).

#### Simple correlation tests using goals as outcome measure

**Fig 1:**
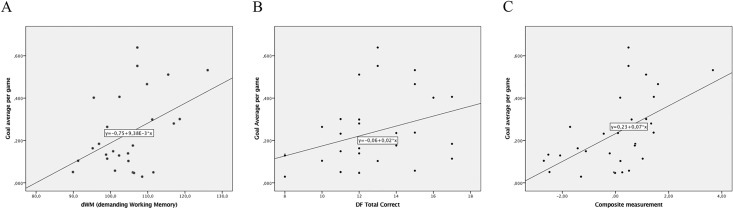
Simple correlation tests using goals as outcome measure for (A) demanding working memory (dWM), (B) Design Fluency Total Correct (DF) and (C) composite measure of the two variables.

***dWM*:** There was a significant correlation between the player’s results on d*WM* and the number of made goals (*r* = .437; *p* = .008; one-tailed correlational test)—see Fig 1A.

***DF*:** There was a significant relationship between the player’s results on *DF Total Correct* and the number of made goals (*r* = .349; *p* = .029; one tailed correlational test)—see Fig 1B.

***Composite measurement*:** There was a significant relationship between the player’s results on the composite measurement, derived from *DF-Total Correct* and *dWM*, and the number of made goals (*r* = .550, *p* = .001; one-tailed correlational test)—see Fig 1C.

#### Partial correlation test using goals as outcome measure

***dWM*:** When controlling for year of birth, length and IQ there was a significant correlation between the player’s results on d*WM* and the number of made goals (*r* = .449; *p* = .009, one-tailed partial correlation test).

***DF*:** When controlling for year of birth, length and IQ there was a significant correlation between the player’s results on *DF-Total Correct* and the number of made goals (*r* = .366; *p* = .030, one-tailed partial correlation test).

***Composite measurement*:** When controlling for year of birth, length and IQ there was a significant relationship between the player’s results on the composite measurement, derived from *DF-Total Correct* and *dWM* and the number of made goals (*r* = .552; *p* = .001, one-tailed partial correlation test).

#### Relation between CWI / TMT and performance

For completeness we performed similar analysis between CWI / TMT and performance as for DF and dWM (See S1 File). In general, we observed weaker results for these tests as predicted, although a moderate relation between CWI 3 and performance was identified.

#### Relation between goals and assists as outcome measure instead of only goals

For completeness we performed similar analysis between a combination score of both goals and assist (instead of only goals) as previously studied in adult soccer players [13] (See S1 File). As predicted we observed similar but weaker results in these analyses.

## Discussion

The present study replicated our previous findings [13] suggesting that general executive functions are important for success in soccer. Moreover, the results were extended from a senior elite level to a junior elite level and shown to be independent from factors such as general intelligence and physical differences. Thereby, the present study has been able to generalize the importance of EF for success in soccer from senior to junior level. The study also suggests that both core EF (CEF) and higher level EF (HEF) are important for soccer success in adolescence—but that only HEF are still developing.

The **cross-sectional tests** showed that the test group was a half SD above the normal population in d*WM*, a test linked to CEF since no cognitive manipulation of information is needed. In *DF-Total Correct*, a test representing HEF as it is associated with several levels of information manipulation, the test group was almost one SD above the normal population. Exploratory analysis of tests that included language-based information, i.e. CWI and TMT showed heterogeneous results. The players performed significant above the normal population in the simpler test TMT2-3 and CWI1-2 but on an average level in TMT-4 and CWI 3. However, since the level of reading ability was not assessed it is hard to interpret the findings on those tests.

The findings above are in agreement with previous studies of adolescents suggesting that young elite soccer players have higher EF capacity than non-elite players [18, 19]—although those studies did not compare the results to a normal population. The two previous studies did not observe any differences between elite players and the semi-elite players concerning simple cognitive processes such as reaction time or basic attentional tasks. In contrast, we observed that the young elite players had significant better capacity of the process-speed and simple attention than the normal population in the present study. Possibly the differences may be explained by different definitions and measurements of reaction-time/process-speed and attention. However, since we observed no relation between simple cognitive processes and performance (Scored goals) our results are in agreement with the previous studies suggesting that these factors are not decisive for success in soccer.

Notably, DF performance results above average of the normal population or above sub-elite level have now been shown in elite soccer players in three independent studies (the present study and [13, 19]). However, in contrast to previous studies on elite adolescent soccer players [18, 19] we also observed a significant effect in the working memory task (dWM). One possibility for this discrepancy may be that our working memory test consisted of a more difficult task since the time periods when the subjects had seen the target could vary unpredictably, mirroring the need of information from different time points on the pitch. Such wide online exploration of different time periods may need a larger degree of EF than in the WM tests used in the other studies.

The **correlational tasks** suggest that both CEF (represented in our study by the encoding skill of the working memory; dWM) and HEF (represented by DF) could be predictive of soccer success in adolescent soccer players in terms of scored goals. This effect was somewhat more robust using a composite measure of the two tests—indicating that the two EF levels both contribute to the behavior. The effects were still present when we controlled for intelligence, length and age in the partial correlation analysis.

The present findings suggest that both HEF and CEF are important components for successful soccer behavior already in adolescence. However, our results suggest that CEF may be relatively more important for soccer success in adolescents than HEF (in comparison with adults). First, the correlation coefficient was moderate for both EF tasks but somewhat stronger for the CEF than for the HEF test in the present sample. Second, when comparing the results from the DF partial correlation tests for young elite players (this sample) and adult elite players (our previous study; [13]) the correlation coefficient is smaller for the young sample suggesting a more important role in adult elite soccer. However, it has to be remembered that the tests were slightly differently performed—for example it is not possible to correct for position for the tested adolescents as the younger players change positions more often than on senior level. Moreover, here we only used goals as our main outcome measure while a combination measurement of goals and assists were used for the adult sample—simply since the adolescent play is less complex and less focused on assists but more focused on scoring goals. In line with this suggestion our exploratory analysis indicated that the results from the partial correlation analysis were not as strong for the composite outcome measure of goals and assists in the present sample (as for the outcome measure of only goals) in line with the idea that assists are more cognitively demanding and require more HEF capacity than observed in children or adolescents. Finally, we also observed that the raw scores of DF improved by age, while the raw scores of dWM did not. Although our sample is small, this suggests that CEF capacity may have reached the maximum development while HEF are still developing in adolescence. Since both EF tasks were proven to be important components for success in soccer this suggests that soccer success in younger individuals is more dependent on CEF than HEF—while HEF becomes gradually more important when approaching adulthood. More specifically, dWM does not contain any cognitive manipulation of the information that is held in the working memory, while DF contains several other on-line multi-processing components such as creativity, response inhibition, and cognitive flexibility [35, 36]. A more complex soccer game on senior elite level requires precisely such information processing.

In the **exploratory analysis** we also observed that the CWI had a strong predictive factor. This was somewhat surprising given that the test group showed only average performance as compared to the norm and that the reading skills may vary among the tested individuals.

The results above make sense considering the maturation of the EF throughout childhood and the adolescence. Different EF abilities develop to their full capacities in different time spans [24, 43]. CEF develop earlier in life and should therefore be more important for contribution to success behaviors in earlier age. Physical ability is important for sport success in all ages. However, we suggest that physical ability is more important in younger age because difference in length, size and strength can be substantial while the play on the soccer-field has not yet become complex. Still our results indicate that the EF are important in early ages as well but on a more basic level. To be successful in goal scoring as a young soccer player it may be more important to process a large amount of information in working memory that can be used to make fast and accurate decisions. When the rules are fixed and the situation is kept constant children display a good capacity in working memory and skill of inhibition [25]. On senior elite level a more complex way of using the EF is needed for successful game behaviors. Greater flexibility related to working memory and inhibition does not reach full maturity until the end of adolescence and in early adulthood [25]. Some brain imaging studies indicate that prefrontal regions mature until the mid-twenties [22]. Possibly, adjustment of behavior for a more accurate and successful outcome is more related to late adolescence while more impulsivity and less accuracy is related to early adolescence.

A main question is whether the above average EF in elite soccer-players are a consequence of practice or represent different genotypes. Top-down regulatory functions such as EF normally vary in the general population depending on the cognitive core capacity in the individual [44]. Although training effects may be present to a certain degree [45], it has been suggested that genetic factors are more important [46]. However, even small improvements may be critical for elite performers in sport. If practice improves EF capacity it may be specifically trained also outside the sport environment. Although it has also been debated whether such cognitive training effects are possible to generalize to other behaviors (i.e. transfer effect) [47, 48], research has suggested that it is possible to train certain cognitive and perceptual skills in different ball sports [49, 50] including soccer [51–54]. More specifically for EF used here, it has been suggested that DF (in contrast to TMT or Stop-signal test) may contain training effects since the better effect of DF in elite players as compared to non-elite players was abolished when weekly training hours had been controlled for [19]. In summary, the causality of above average EF observed in elite soccer players is not yet established. We suggest that although cognitive training may improve EF that are used in soccer and may be complement for elite soccer players, it is even more important to find players with the right cognitive profile from the start. Also, while it is interesting to better understand the effect of training cognitive functions such as EF on soccer performance, it is first important to establish a strong relation between EF and sport success.

Although at least four studies including the present [14, 18, 19] suggests that above average EF are important for soccer in general, this model is probably over-simplified. It is more likely that different cognitive profiles are important for different playing positions. While midfielders may need stable EF over long time, attackers may need more impulsivity and defenders more inhibitory functions. Future studies will have to more specifically study different successful cognitive profiles in soccer related to different playing positions. This knowledge may be especially interesting for adolescents since this is the period when the individual player is trying out various positions that he or she will specialize for.

## Supporting information

S1 File 1) A more detailed description of the testing procedures. 2) Normative data and Reliability / Validity for D-KEFS and CogStateSport. 3) Results from testing the relation between CWI / TMT and performance. 4) Results using goals and assists as outcome measure instead of only goals. 5) Results from partial correlation tests using goals and assists as outcome measure (dWM/DF/CWI/TMT). 6) Supplemental Figures showing age distribution of the participants by year of birth and by age.
(DOCX)
