# Supplementary material for: Core executive functions are associated with success in young elite soccer players
Source: PLoS One. 2017 Feb 8;12(2):e0170845. doi: 10.1371/journal.pone.0170845 (PMC5298906; doi:10.1371/journal.pone.0170845)
Supplement: S1 File — 1) A more detailed description of the testing procedures. 2) Normative data and Reliability / Validity for D-KEFS and CogStateSport. 3) Results from testing the relation between CWI / TMT and performance. 4) Results using goals and assists as outcome measure instead of only goals. 5) Results from partial correlation tests using goals and assists as outcome measure (dWM/DF/CWI/TMT). 6) Supplemental Figures showing age distribution of the participants by year of birth and by age. (DOCX) [file pone.0170845.s001.docx]

**Supplementary materials**

**Core executive functions are associated with success in young elite soccer players**

**Vestberg T, Reinebo G, Maurex L, Ingvar M, Petrovic P**

**The test procedure**

The test sessions lasted approximately one hour per subject. The participating players were tested one by one in an office room at a facility next to the academies training ground from June till October 2013. The session started with *TMT* and *DF* followed by the *Cogsport* test battery. After *Cogsport* the subjects were tested on *CWI*. The session was ended with *Raven’s Standard Progressive Matrices*. All subjects had the same test leader.

**Normative data and Reliability/Validity of the used tests**

**The Normative data for D-KEFS**

**D-KEFS** [1] is used world wide for testing capacity of EF in clinical assessments and in research. The standardization sample included 1750 children, adolescents and adults (8 to 89 years of age) [2]. The 2000 US Census figures were used as target values for the normative data. The selected sample was based on age, gender, socioeconomic factors etc. The normative samples are divided into age groups. Thus, every individual that is tested using D-KEFS is compared to a large group of individuals of the same age span. The reliability and validity for D-KEFS has been considered as good [2, 3]. The test re-test reliability for the tests used in the present study is shown below - extracted from [2].

**The test-retest reliability for D-KEFS, age 8 - 19**

**TMT 2-3**, *Combined numbers and letters*, *r* = 0.78

**TMT 4**, *Switching*, *r* = 0.20.

The lower result in TMT 4, compared to TMT 2-3, is due to an improvement of the test performance in the second test (suggesting learning effects).

**DF**, *Condition 1*, *r* = 0.66

**DF**, *Condition 2*, *r* = 0.43

**DF,** *Condition 3*, *r* = 0.13

The lower result in Condition 3 compared with Condition 1 and 2 is due to an improvement of the test performance in the second test (suggesting learning effects).

**CWI**, Condition 1, *r* = 0.79

**CWI,** Condition 2, *r* = 0.77

**CWI,** Condition 3, *r* = 0.90

**CWI**, Condition 4, *r* = 0.80

The results were in general higher for all subjects in the second testing.

**The Normative data for CogStateSport**

The original normative data is based on 300 young adults in Australia [4]. Additional baseline data from collaborative studies around the world has been added subsequently. The sample size for each of the age is for the moment:

Age n

12 2634

13 3423

14 9468

15 9785

16 8345

17 7588

18 3679

19 1475

The sample-size data above is reported from the research and development department of CogStateSport (personal communications). Thus, every individual that is tested using CogStateSport is compared to a large group of individuals of the same age span. The clinical validity for CogStateSport has been shown for head injured athletes [4] and the test-retest reliability has been considered fairly good [4], including for dWM (see below). Another recently published study on adults showed that the test-retest reliability for dWM *(Learning)* was high [5].

**The test-retest reliability for dWM / CogStateSport** [4]

**(dWM)** *Learning*, speed, one hour, ICC (intraclass correlation coefficient) = 0.83

**(dWM)** *Learning*, speed, one week, ICC = 0.82

**(dWM)** *Learning*, accuracy, one hour**,** ICC = 0.45

**(dWM)** *Learning*, accuracy, one week**,** ICC = 0.47

**Exploratory analyses**

**Section A: Testing the relation between CWI / TMT and performance**

**Cross-sectional tests (CWI/TMT):**

***CWI*:** The soccer players performed 0.26 SD above the normal population in average in the *Combined condition 1-2* scaled scores (t(29) = 3.096, *p* < .004; two-tailed) The soccer players did not perform significantly different as compared to the normal population (+ 0.02 SD; normed mean: 10; SD = 3) in *Condition 3* (t(29) = 0.844, *p* > .05; two-tailed).

***TMT*:** The soccer players performed 1.14 SD above the normal population in average in the *Combined condition 2-3* scaled scores (t(29) = 10.058, *p* < .000; two-tailed) but not significantly different as compared to the norm in *Condition 4* scaled scores ((- 0.21 SD; t(29) = -1.279, *p* > .05; two-tailed).

**Simple correlation tests using goals as outcome measure (CWI/TMT):**

**CWI:** There was no significant relationship between the player’s results on *CWI 1-2* and the number of made goals (*r* = -.003; *p* = .493; one-tailed correlational test). There was a significant relationship between the player’s results on *CWI-3* and the number of scored goals (*r* = .435; *p* = .008, one tailed correlational test).

**TMT:** There was no significant relationship between the player’s results on *TMT 2-3* and the number of made goals (*r* = .009; *p* = .480; one-tailed correlational test) nor was there any significant relationship between the player’s results on *TMT 4* and the number of scored goals (*r* = .04; *p* = .416; one- tailed correlational test).

**Partial Correlation test using goals as outcome measure (CWI/TMT):**

**CWI:** When controlling for year of birth, length and IQ of the players there was no significant relationship between the player’s results on *CWI 1-2* and the number of scored goals (*r* = .015; *p* = .470, one-tailed partial correlation test) When controlling for year of birth, length and IQ there was a significant correlation between the player’s results on *CWI-3* and the number of made goals (*r* = .465; *p* = .007, one-tailed partial correlation test).

**TMT:** When controlling for year of birth, length and IQ of the players there was no significant relationship between the player’s results on *TMT 2-3* and the number of scored goals (*r* = .016; *p* = .468, one-tailed partial correlation test) nor any significant relationship between the player’s results on *TMT 4* and the number of made goals (*r* = .038; *p* = .426, one-tailed partial correlation test).

**Section B: Using goals and assists as outcome measure instead of only goals**

**Simple correlation tests using goals and assists as outcome measure (dWM/DF/CWI/TMT):**

**dWM:** There was a non-significant trend effect for a correlation between the player’s results on d*WM* and the number of goals/assist (*r* = .276; *p* = .070 one-tailed Pearsons correlational test).

**DF:** There was no significant relationship between the player’s results on *DF Total Correct* and the number of goals/assist (*r* = .175; *p* = .178; one-tailed correlational test).

**Composite measurement (dWM/DF):** There was a significant relationship between the player’s results on the composite measurement, derived from *DF-Total Correct* and *dWM*, and the number of goals/assists (*r* = .316, *p* = .045; one-tailed correlational test).

**CWI:** There was no significant relationship between the player’s results on *CWI 1-2* and the number of goals/assist (*r* = .099; *p* = .301, one-tailed correlational test). There was a significant relationship between the player’s results on *CWI-3* and the number of goals/assist (*r* = .440; *p* = .007; one-tailed correlational test).

**TMT:** There was no significant relationship between the player’s results on *TMT 2-3* and the number of goals/assist (*r* = .095; *p* = .309, one-tailed correlational test) nor between the player’s results on *TMT 4* and the number of goals /assist (*r* = .048; *p* = .401, one-tailed correlational test).

**Partial Correlation test using goals and assists as outcome measure (dWM/DF/CWI/TMT):**

**dWM:** When controlling for year of birth, length and IQ there was a significant relationship between the player’s results on d*WM* and the number of goals/assist (*r* = .332; *p* = 0.045; one-tailed partial correlation test).

**DF:** When controlling for year of birth, length and IQ there was no significant relationship between the player’s results on *DF (Total Correct)* and the number of goals/assist (*r* = .188; *p* = .174, one-tailed partial correlation test).

**CWI:** When controlling for year of birth, length and IQ there was no significant relationship between the player’s results on *CWI 1-2* and the amount of goals/assist (*r* = .061; *p* = .381, one-tailed correlational test). When controlling for year of birth, length and IQ there was a significant relationship between the player’s results on *CWI-3* and the number of goals/assist (*r* = .429; *p* = .013, one-tailed partial correlation test).

**TMT:** When controlling for year of birth, length and IQ there was no significant relationship between the player’s results on *TMT 2-3* and the amount of goals/assist (*r* = .124; *p* = .268, one-tailed partial correlation test) nor any significant relationship between the player’s results on *TMT 4* and the number of goals/assist (*r* = .025; *p* = .452, one-tailed partial correlation test).

**Composite measure (dWM/DF):** When controlling for year of birth, length and IQ there was a significant relationship between the player’s results on the composite measurement, derived from *DF-Total Correct* and *dWM,* and the number of goals/assist (*r* = .351, *p* = .036, one-tailed partial correlation test).

**Controlling for Month of birth**

When controlling for month of birth it didn’t make any real influence on the correlation between the player’s results on *dWM* and the amount of goals they made between 2012 and 2014.

Controlling for birth month, r = .438; p < .009 (one-tailed).

Not controlling for birth month, r = .437; p < .008 (one-tailed).

When controlling for the month of birth it didn’t make any real influence on the correlation between the player’s results on *DF-Total Corrected* and the amount of goals they made between 2012 and 2014.

Controlling for birth month, r = .349; p < .032 (one-tailed).

Not controlling for birth month, r = .349; p < .029 (one-tailed).

There was no significant relationship between the player’s month of birth and the amount of goals they made 2012 and 2014, r = -.011; p > .05 (one tailed).

**Supplemental Figures**


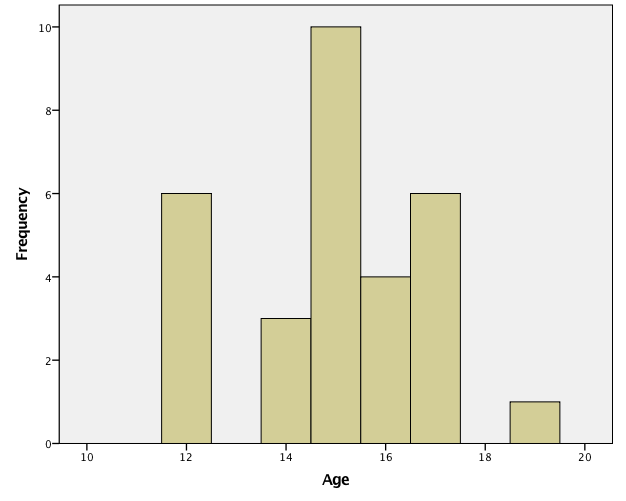


**S1 Fig A.** Age distribution of the participants by age (n = 30).


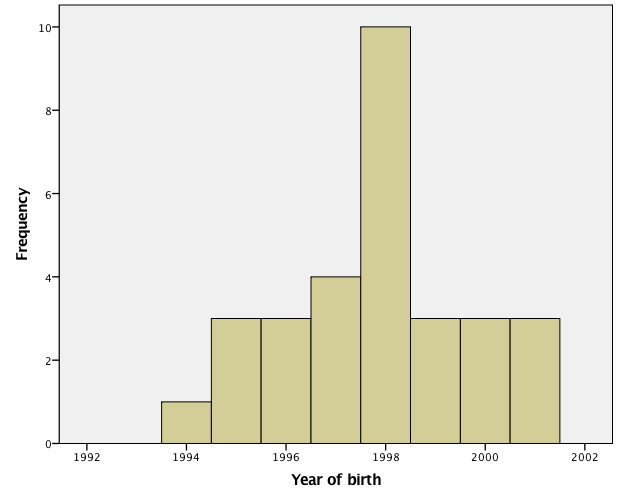


**S1 Fig B.** Age distribution of the participants by year of birth (n = 30).

**References (Supplementary materials):**

1. Delis DC, Kaplan E, Kramer JH. Delis-Kaplan Executive Function System (D-KEFS) examiner’s manual. San Antonio, Texas, USA: The Psychological Corporation; 2001a.
2. Delis DC, Kaplan E, Kramer JH. Delis-Kaplan Executive Function System (D-KEFS) technical manual. San Antonio, Texas: The Psychological Corporation; 2001b. 1-132.
3. Homack S, Lee D, Riccio CA. Test review: Delis-Kaplan executive function system. Journal of clinical and experimental neuropsychology. 2005;27(5):599-609.
4. Collie A, Maruff P, Makdissi M, McCrory P, McStephen M, Darby D. CogSport: reliability and correlation with conventional cognitive tests used in postconcussion medical evaluations. Clinical Journal of Sport Medicine. 2003;13(1):28-32.
5. Louey AG, Cromer JA, Schembri AJ, Darby DG, Maruff P, Makdissi M, et al. Detecting cognitive impairment after concussion: sensitivity of change from baseline and normative data methods using the CogSport/Axon cognitive test battery. Archives of clinical neuropsychology : the official journal of the National Academy of Neuropsychologists. 2014;29(5):432-41.
